# Vitamin D Status, Disease Activity, and Endothelial Dysfunction in Early Rheumatoid Arthritis Patients

**DOI:** 10.1155/2017/5241012

**Published:** 2017-10-22

**Authors:** Alexandru Caraba, Viorica Crişan, Ioan Romoşan, Ioana Mozoş, Marius Murariu

**Affiliations:** ^1^Department of Internal Medicine, Division of Rheumatology, University of Medicine and Pharmacy “Victor Babeş”, Timişoara, Romania; ^2^Division of Rheumatology, Timişoara, Romania; ^3^Department of Internal Medicine, University of Medicine and Pharmacy “Victor Babeş”, Timişoara, Romania; ^4^Department of Pathophysiology, University of Medicine and Pharmacy “Victor Babeş”, Timişoara, Romania; ^5^University of Medicine and Pharmacy “Victor Babeş”, Timişoara, Romania

## Abstract

Cardiovascular diseases represent important complications in rheumatoid arthritis (RA) patients, generated by an accelerated atherosclerosis. The aim of this study is represented by the assessment of the correlations between serum levels of vitamin D, disease activity, and endothelial dysfunction in patients with early RA. *Material and Methods*. The study was performed on a group of 35 patients with early RA and 35 healthy subjects matched for age and gender, as controls. In all studied subjects, the following were determined: inflammatory markers, insulin resistance, vitamin D levels, and endothelial dysfunction. Statistical analysis were performed using the Student's *t*-test and the Pearson's test. *p* values of less than 0.05 were considered statistically significant. *Results*. The group of patients with RA patients presented inflammation, low levels of vitamin D, elevated insulin resistance, and reduced flow-mediated vasodilation, statistically significant compared to the control group (*p* < 0.00001). Significant inverse correlations between the levels of 25(OH) vitamin D and DAS28, respective insulin resistance, and significant positive correlation between 25(OH) vitamin D and endothelial function were demonstrated. *Conclusion*. In early RA patients with moderate and high disease activity, low serum levels of vitamin D were associated with disease activity, increased insulin resistance, and endothelial dysfunction.

## 1. Introduction

Rheumatoid arthritis (RA) is a chronic inflammatory autoimmune disorder, which typically involves small- and medium-sized joints. Rheumatoid synovitis generates cartilage breakdown, bony erosions, and loss of function of the involved joints [1]. But besides articular involvement, cardiovascular disease generated by accelerated, premature atherosclerosis represents a serious complication of RA. It is known that in RA patients, cardiovascular disease represents the cause of 40–50% of the deaths in this group of population [2, 3]. Atherosclerosis is a complex process, which develops over the course of many years, beginning in the early teenage years, and endothelial dysfunction represents the first step in its development [4, 5].

Lin et al. showed in their meta-analysis that the RA patients exhibit lower vitamin D levels than healthy controls, and, on the other hand, these levels present an inverse correlation with the disease activity [6]. Other studies demonstrated the role of vitamin D in RA activity [7–10]. Besides the role in bone mineral metabolism, vitamin D has anti-inflammatory and immunomodulatory roles. By antiproliferative, antiangiogenic, and antioxidant properties, vitamin D offers protective effects on the cardiovascular system [11, 12].

The aim of this study is represented by the assessment of the associations between serum levels of vitamin D, disease activity, and endothelial dysfunction in patients with early rheumatoid arthritis.

## 2. Material and Methods

### 2.1. Patients

The study was performed on a group of 35 patients with early RA and 35 healthy subjects matched for age and gender, as controls. The diagnosis of RA was established based on the 2010 American College of Rheumatology/European League Against Rheumatism Classification Criteria for Rheumatoid Arthritis [13]. All patients had a disease duration of less than 2 years. Exclusion criteria were as follows: previous steroid therapy or drugs that alter insulin sensitivity, diabetes mellitus, uncontrolled arterial hypertension, dyslipidemia, chronic kidney disease, thyroid dysfunction, Cushing's syndrome, current smokers, patients with history of acute coronary syndrome during the last 6 months, pregnancy, and patients taking vitamin D replacement therapy. All patients gave their informed consent. The study was approved by the Ethics Committee of University of Medicine and Pharmacy “Victor Babeş” Timişoara, Romania.

### 2.2. Methods

In all patients, the following were determined: anti-citrullinated peptide antibodies (chemiluminescent microparticle immunoassay, serum), rheumatoid factor (turbidimetry, serum), erythrocyte sedimentation rate (ESR) (Electro Optical System Technologies), C-reactive protein (turbidimetry, serum), fibrinogen (coagulation, plasma citrate), TNF-*α* (chemiluminescence immunoassay, serum), IL-6 (electrochemiluminescence immunoassay, serum), fasting insulinemia (chemiluminescence immunoassay, serum) and glycemia (photometry, plasma NaF K2 oxalate), and vitamin D levels (25(OH) vitamin D) (chemiluminescence immunoassay, serum).

Rheumatoid arthritis activity was assessed using Disease Activity Score 28 (DAS28). DAS28 was calculated based on ESR, tender joint count (28 joints), swollen joint count (28 joints), and the patient's assessment of global well-being (100 mm visual analogue scale) (http://www.4s-dawn.com/DAS28/DAS28.html).

Insulin resistance was assessed by homeostasis model assessment of insulin resistance (HOMA-IR) index, using fasting insulin and glucose [14].

Endothelial dysfunction was assessed by means of flow-mediated vasodilation, on brachial artery, using B-mode ultrasonography (Siemens Acuson X300 Ultrasound System, with linear transducer of 10 MHz). Before the test, the patient was relaxed at a stable room temperature between 20–25°C; ingestion of caffeine, high-fat foods, and vitamin C was prohibited. The diameter of the brachial artery was measured incidentally with the *R* wave of the electrocardiograph trace (Di). Then, ischemia was induced by inflating the pneumatic cuff to a pressure 50 mmHg above systolic one, in order to obliterate the brachial artery and induce ischaemia. After 5 minutes, the cuff was deflated and the diameter was measured after 60-second postdeflation (Df). FMD was calculated with the formula: FMD = [(Df − Di)/Di] × 100 [15].

In controls, the following were determined: erythrocyte sedimentation rate (ESR), C-reactive protein, fibrinogen, TNF-*α*, IL-6, fasting insulinemia and glycemia, vitamin D levels, insulin resistance, and flow-mediated vasodilation, using the same methods.

### 2.3. Statistical Analysis

Data were expressed as mean ± standard deviation. Statistical analyses were performed using the Student's *t*-test and Pearson's correlation. Differences were considered statistically significant at the value of *p* < 0.05.

## 3. Results

The demographic data of patients and controls are presented in Table 1.

All patients were positive for rheumatoid factor and anti-citrullinated peptide antibodies.

The laboratory findings of RA patients and controls are presented in Table 2.

By analyzing these data, it can be observed that all RA patients presented inflammation, low levels of vitamin D, elevated insulin resistance, and reduced flow-mediated vasodilation. These parameters showed statistically significant differences between RA patients and controls, as presented in Table 2.

Among the RA patients, only one had vitamin D insufficiency, while another 34 patients had vitamin D deficiency.

Based on DAS28 value, the RA patients were divided into two subgroups: one with moderate disease activity and the other with high disease activity. The RA patients with high disease activity presented lower values of FMD and 25(OH) vitamin D and higher values of HOMA-IR, compared with the RA patients with moderate disease activity (Table 3).

The results of this study showed that the entire group of patients with early active RA presented low levels of 25(OH) vitamin D, high insulin resistance, and endothelial dysfunction.

**Figure 1:**
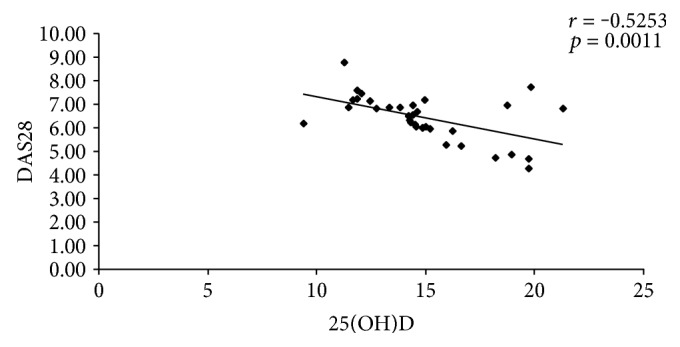
Correlation between 25(OH)D and DAS28.

There were demonstrated significant inverse correlations between the levels of 25(OH) vitamin D and DAS28 (*p* = 0.0011), and respective insulin resistance (*p* = 0.0389), and significant positive correlation between 25(OH) vitamin D and endothelial function, expressed as FMD (*p* = 0.0010). The same correlations were identified in the two subgroups of patients with moderate disease activity and respective high disease activity (Table 4, Figures 1(), 2(), and 3()).

Studying the correlations between proinflammatory cytokines and the levels of 25(OH) vitamin D, significant inverse correlations were identified between 25(OH) vitamin D and TNF-*α* (*r* = −0.4269, *p* = 0.0105), respective IL-6 (*r* = −0.3627, *p* = 0.0322).

On the other hand, high disease activity of RA, expressed as DAS28, has been positively correlated with insulin resistance (*r* = 0.3692, *p* = 0.0029) and negatively correlated with FMD (*r* = −0.3912, *p* = 0.0020).

## 4. Discussion

Rheumatoid arthritis affects up to 1% of adults worldwide, representing a serious public health problem, because of articular and extra-articular involvement. Morbidity and mortality due to atherosclerotic cardiovascular diseases are increased in RA patients [16, 17]. Based on the studies of Meune et al. and Avina-Zubieta et al., RA is associated with a 1.48-fold increase in atherosclerotic cardiovascular diseases and a 1.6-fold increase in cardiovascular disease-related death, compared to the general population [18, 19]. Atherosclerotic cardiovascular disease in RA patients has more severe presentation and worse outcomes compared to the general population [20]. Traditional cardiovascular risk factors and RA-related risk factors contribute to this excess of cardiovascular morbidity and mortality of these patients. Among RA-related risk factors, proinflammatory cytokines (TNF-*α*, IL-6), oxidative stress, an increase of leptin and resistin (proatherogenic hormones) and the decrease of adiponectin (antiatherogenic hormone), and insulin resistance play the most important role in accelerated atherogenesis. The first step in the atherosclerosis process is represented by the endothelial dysfunction [4, 19, 21].

In the last couple of years, it has been emphasized the role of vitamin D in health and disease. Besides the well-known effects on bone metabolism, vitamin D has effects on the immune and cardiovascular systems [22–24].

Normal levels of vitamin D are required to maintain the physiological innate and adaptive immune responses and the immune tolerance of self-antigens. Vitamin D deficiency is associated with the loss of immune tolerance and the appearance of autoimmunity processes, including rheumatoid arthritis [16, 22, 25–27]. The protective effects of vitamin D on the cardiovascular system are represented by the increase of anti-inflammatory cytokine expression (such as IL-10) and by the decrease of proinflammatory molecule expression (such as TNF-*α* and IL-6) [28].

The connections between low levels of vitamin D, inflammation, insulin resistance, and endothelial dysfunction in rheumatoid arthritis are very complex.

Accelerated atherosclerosis associated with RA is related to systemic inflammation, which characterized this disease [29]. Systemic inflammation contributes to the initiation and development of accelerated atherosclerosis, since inflammatory processes in the rheumatoid synovium and atherosclerotic plaques are remarkably similar. Inflammatory response acts in endothelial dysfunction appearance by means of proinflammatory cytokines, such as TNF-*α*, IL-6, and IL-1 [30]. In RA, low levels of vitamin D are noted to be common and are even more prevalent than in the general population. These low levels are described to be associated with some cardiovascular risk factors [2, 12, 31]. But vitamin D deficiency is associated with an exacerbation of Th1 immune response, resulting in the upregulation of the expression and production of several proinflammatory cytokines including TNF-*α*, IL-1*β*, IL-6, and IL-8, too [32–34]. Inflammatory responses play a crucial role in the pathogenesis and development of IR [35], and the low levels of 25(OH) vitamin D are associated with IR [36]. Insulin resistance induces vasoconstriction and vascular smooth muscle cell proliferation, generating endothelial dysfunction [37].

In the present study, the levels of 25(OH) vitamin D were decreased in all studied patients, compared with controls (*p* < 0.00001), as it has been shown in other studies [2, 12, 31]. Significant inverse correlations (*p* = 0.0105 for TNF-*α*, respective *p* = 0.0322 for IL-6) have been highlighted between the levels of proinflammatory cytokines (TNF-*α* and IL-6) and the levels of 25(OH) vitamin D. Welsh et al. showed that the degree of systemic inflammation is inversely associated with the circulating levels of vitamin D [31].

Vitamin D may also have a role in modulating RA disease activity [7]. The results of the present study showed that the low levels of 25(OH) vitamin D were correlated in an inversely manner with the RA activity, expressed as DAS28 (*p* = 0.0011). The same inverse correlations were identified in RA patients with moderate disease activity (*p* = 0.046) and respective high disease activity (*p* = 0.0397). Rossini et al., studying 1191 RA patients and 1019 healthy controls, identified a significant inverse correlation between the logarithm of the 25(OH) vitamin D levels and the RA activity (*p* = 0.002) [38]. Hong et al. demonstrated on the 130 patients with RA and 80 healthy controls that the RA patients had lower levels of 25(OH) vitamin D and these levels correlated significantly with the RA activity (*r* = −0.43) [16]. El-Barbary et al. included in their study forty early RA patients and forty healthy controls. The authors demonstrated the significant negative correlation between 25(OH) vitamin D levels and DAS28, respective IL-6 (*p* < 0.001) [39]. Other studies obtained the same relationship between the levels of serum 25(OH) vitamin D and disease activity in RA patients, reporting the correlation coefficient as *r* = −0.57 [40], *r* = −0.431 [41], *r* = −0.42 [42], and *r* = −0.604 [43].

Hypovitaminosis D increases the insulin resistance and may trigger endothelial dysfunction [44]. In our study, it was identified a significant inverse correlation between low levels of serum 25(OH) vitamin D and insulin resistance, expressed as HOMA-IR (*p* = 0.0389). Strong inverse correlation was obtained in both subgroups of rheumatoid patients with moderate disease activity (*p* = 0.009) and respective high disease activity (*p* = 0.0396). Hirschler et al. reported the same results in their study (*r* = −0.29, *p* = 0.002) [45]. The role of vitamin D in modulating insulin sensitivity is emphasized in the study published by Miñambres et al. [46].

Previous studies have shown that insulin resistance is associated with endothelial dysfunction, an early marker of atherosclerosis [21, 47, 48].

Borges et al. demonstrated that vitamin D improved the endothelial function, suggesting the relationship between hypovitaminosis D and endothelial dysfunction [49]. The present study showed the positive correlation between the serum levels of 25(OH) vitamin D and endothelial function expressed as FMD (*p* = 0.0010). In the subgroup of RA patients with moderate disease activity, it was shown a positive correlation between the serum levels of 25(OH) vitamin D and endothelial function (*p* = 0.049), while in a subgroup of patients with high disease activity, the correlation was positive, too, but more significant (*p* = 0.001). The explanation of this finding is based on the fact that high disease activity is associated with lower levels of 25(OH) vitamin D, higher inflammatory responses and insulin resistance, and significant endothelial dysfunction [35–37]. In their study, Jablonski et al. found endothelial dysfunction in adults with hypovitaminosis D, as opposed to patients with normal levels of this vitamin [50]. In RA patients with vitamin D deficiency, Ranganathan et al. revealed a significant correlation between serum 25(OH) vitamin D and endothelial function (*p* = 0.04) [51].

Our study revealed that low levels of 25(OH) vitamin D were associated with high disease activity, high insulin resistance, and endothelial dysfunction in early RA patients. But disease activity in itself contributes to the development of atherosclerosis through insulin resistance [52–54]. Between DAS28, as a marker of RA activity and FMD, it was observed as an inverse correlation (*r* = −0.3912, *p* = 0.020), and, on the other hand, between DAS28 and insulin resistance it was identified as a positive significant correlation (*r* = 0.3692, *p* = 0.029).

The present study had some limitations. The first limitation was the small sample size, being investigated only 35 patients. Second, only the serum 25(OH)D was determined. The free bioavailable vitamin D and vitamin D-binding protein were not measured.

## 5. Conclusion

In early RA patients with moderate and high disease activity, low serum levels of vitamin D were associated with disease activity, increased insulin resistance, and endothelial dysfunction.

## Figures and Tables

**Figure 2:**
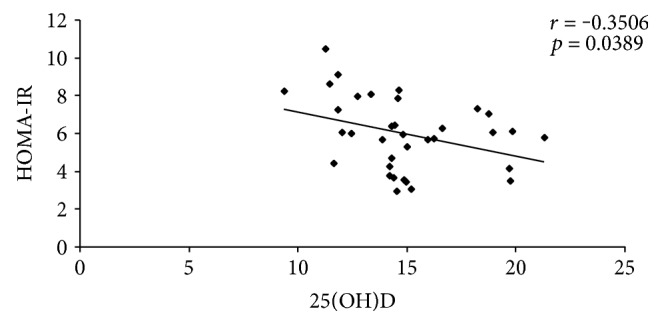
Correlation between 25(OH)D and HOMA-IR.

**Figure 3:**
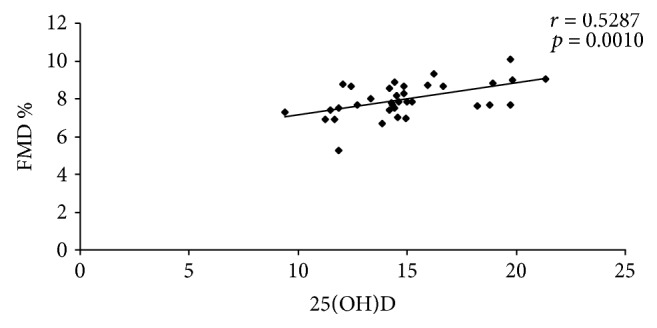
Correlation between 25(OH)D and FMD%.

**Table 1:** Demographic data in RA patients and controls.

Parameter	Value (mean ± standard deviation)	
	RA patients	Controls
Sex [*n* (%)]	35	35
Males	12 (34.28%)	12 (34.28%)
Females	23 (65.72%)	23 (65.72%)
Mean age (years)	55.6 ± 9.74	54.14 ± 6.28
Mean length of RA evolution (months)	14.25 ± 5.27	—
The drugs used by the RA patients in the moment of investigation	Methotrexate (30 patients; 13.28 ± 2.25 mg/week) and leflunomide (5 patients; 20 mg/day)	—

**Table 2:** Laboratory findings in RA patients and controls.

Parameter	Value (mean ± standard deviation)		*p*
	RA patients	Controls	
ESR (mm/h)	74.11 ± 18.47	8.45 ± 2.99	<0.00001
C-reactive protein (mg/l)	60.34 ± 27.8	2.88 ± 0.98	<0.00001
Fibrinogen (mg/dl)	693.52 ± 284.61	207.18 ± 95.24	<0.0001
DAS28	6.41 ± 0.94	—	—
TNF-*α* (pg/ml)	89.65 ± 21.41	3.76 ± 1.64	<0.00001
IL-6 (pg/ml)	90.15 ± 20.79	4.41 ± 1.78	<0.00001
FMD (%)	7.94 ± 0.81	13.78 ± 1.43	<0.00001
HOMA-IR	5.97 ± 1.89	1.23 ± 0.19	<0.00001
25(OH) vitamin D	14.90 ± 2.81	36. 39 ± 7.78	<0.00001

**Table 3:** Differences of FMD, HOMA-IR, and 25(OH) vitamin D between moderate and high disease activity RA patients.

Parameter	Disease activity		*p*
	Moderate	High	
Number of patients	4	31	
DAS28	4.63 ± 0.26	6.64 ± 0.73	<0.0001
FMD (%)	8.88 ± 0.88	7.23 ± 0.82	<0.05
HOMA-IR	4.27 ± 1.23	6.13 ± 1.92	<0.05
25(OH) vitamin D	19.15 ± 0.72	14.35 ± 2.49	<0.0001

**Table 4:** Correlations between 25(OH) vitamin D and disease activity, insulin resistance, and endothelial function in RA patients.

Correlation	RA patients		
	Whole group	Moderate disease activity group	High disease activity group
25(OH) vitamin D-DAS28	*r* = −0.5253	*r* = −0.9072	*r* = −0.3198
	*p* = 0.0011	*p* = 0.046	*p* = 0.0397
25(OH) vitamin D-HOMA-IR	*r* = −0.3506	*r* = −0.9813	*r* = −0.3200
	*p* = 0.0389	*p* = 0.009	*p* = 0.0396
25(OH) vitamin D-FMD	*r* = 0.5287	*r* = 0.9001	*r* = 0.5144
	*p* = 0.0010	*p* = 0.049	*p* = 0.001
